# Draft Genome Sequence of *Streptomyces roseochromogenes* subsp. *oscitans* DS 12.976, Producer of the Aminocoumarin Antibiotic Clorobiocin

**DOI:** 10.1128/genomeA.01147-13

**Published:** 2014-01-09

**Authors:** Christian Rückert, Jörn Kalinowski, Lutz Heide, Alexander Kristian Apel

**Affiliations:** Technology Platform Genomics, CeBiTec, Bielefeld University, Bielefeld, Germanya; Pharmaceutical Institute, Tübingen University, Tübingen, Germanyb

## Abstract

*Streptomyces roseochromogenes* subsp. *oscitans* DS 12.976 is the producer of the gyrase-inhibiting aminocoumarin antibiotic clorobiocin. Here, we present a draft genome sequence of this strain, in which we identified the clorobiocin gene cluster as well as an unusually high number (43) of further putative secondary metabolite clusters.

## GENOME ANNOUNCEMENT

*Streptomyces roseochromogenes* subsp. *oscitans* DS 12.976 (NRRL 3504) was described as a producer of the aminocoumarin antibiotic clorobiocin (1), a potent gyrase inhibitor binding to the GyrB subunit of bacterial gyrase (2). The structurally very similar aminocoumarin antibiotic novobiocin has been in clinical use under the name Albamycin and is still used in veterinary medicine. The strain NRRL 3504 is clearly different from *S. roseochromogenes* DSM 40463, with which it shares only 96% of its nucleotides on the 16S rRNA level (locus GU383178). It is most closely related to the *Streptomyces* strains *Streptomyces* sp. NEAU-YX9, *Streptomyces ginsengisoli*, and *Streptomyces durhamensis* NRRL B-3309, sharing 99% 16S rRNA identity.

Here, we present its draft genome sequence, in which we identified the clorobiocin gene cluster that has been described previously (1) with the help of the program antiSMASH (3).

Forty-three further gene clusters for the biosynthesis of secondary metabolites were identified (1 melanin, 3 siderophore, 1 mixed siderophore/bacteriocin, 1 mixed siderophore/polyketide synthetase [T1-PKS], 8 terpene, 1 mixed terpene/melanin, 4 polyketide synthetase [2 T1-PKS, 1 T3-PKS, 1 T4-PKS], 2 butyrolactone, 1 ectoine, 4 bacteriocin, 3 nonribosomal peptide synthetase [NRPS], 2 mixed NRPS/bacteriocidin, 2 mixed NRPS/polyketide synthetase [1 T2-PKS, 1 T1+T3-PKS], 2 lantipeptide, and 8 unspecified clusters).

The genomic sequence of *S. roseochromogenes* was obtained by assembly of two data sets, which were generated by paired-end and whole-genome shotgun pyrosequencing strategies (4) utilizing the Newbler software (version 2.5.3). Overall, 688,633 reads were assembled to an initial draft genome of 9,765,775 nucleotides at 20.39-fold coverage. The resulting draft genome sequence consists of 433 contigs (385 contigs contain >500 bases) in 12 scaffolds, with an overall G+C content of 70.55%. Manual *in silico* assembly revealed the presence of a linear plasmid, pSros1, with a size of 119.0 kb. The final draft sequence consists of two scaffolds, one for the linear chromosome and one for the plasmid, consisting of 397 contigs (9.66 Mbp; chromosome) and 5 contigs (119.0 kbp; pSros1), respectively. The assembled contigs were annotated with the PGAAP pipeline (5), resulting in the annotation of 8,942 coding sequences (CDSs). Furthermore, we identified 6 rRNA operons and 68 tRNA loci.

The *S. roseochromogens* subsp. *oscitans* DS 12.976 genome sequence will allow for the regulation of clorobiocin biosynthesis to be investigated and for new gene clusters and bioactive compounds to be mined.

### Nucleotide sequence accession numbers.

This whole-genome shotgun sequence has been deposited in DDBJ/EMBL/GenBank under the accession no. AWQX00000000. The version described in this paper is version AWQX01000000.
